# Oral Health of Children One to Six Years after Dental Treatment under General Anaesthesia

**DOI:** 10.3390/jcm11133676

**Published:** 2022-06-25

**Authors:** Vicky Ehlers, Angelika Callaway, Michael Patyna, Alexandra Pelkner, Birgül Azrak, James Deschner

**Affiliations:** Department of Periodontology and Operative Dentistry, University Medical Center of the Johannes Gutenberg University Mainz, D-55131 Mainz, Germany; angelikascallaway@aol.com (A.C.); michael.patyna@unimedizin-mainz.de (M.P.); a.pelkner@web.de (A.P.); b.azrak@dr-azrak.de (B.A.); james.deschner@uni-mainz.de (J.D.)

**Keywords:** early childhood caries, dental treatment under general anaesthesia, oral health, questionnaire

## Abstract

(1) Background: The aim of this study was to assess oral health in children following dental treatment under general anaesthesia and to obtain information about oral health measures in both the children and their parents. (2) Methods: Children were scheduled for regular dental re-examination one to six years after dental treatment under general anaesthesia. Scores for mixed dmft/DMFT, the plaque control record (PCR), and the gingival bleeding index (GBI) were determined. Information about children’s/parents’ oral hygiene habits and frequency of dental visits was obtained. Scores for mixed dmft/DMFT as assessed before dental treatment under general anaesthesia/at re-examination were compared (Wilcoxon test). (3) Results: From the 134 parents initially contacted, 35 attended regular dental control visits (response rate: 26%) with their children (median age 6 years). Of the 35 children (20 female, 15 male), 18 (51.4%) were healthy and 17 (48.6%) had a pre-existing condition. Mixed dmft/DMFT scores determined at the recall visit differed significantly from the earlier visit (*p* = 0.006). Children had 1.74 ± 3.64 teeth newly affected by caries. Four children (11.4%) needed dental treatment under general anaesthesia again. Oral hygiene was mediocre (median PCR: 32%). The GBI was high (median: 14%). Children with a high PCR also had a high GBI. (4) Conclusions: Children who had received dental treatment under general anaesthesia still had a high caries risk. Further prophylaxis programs are necessary to prevent caries and further use of general anaesthesia.

## 1. Introduction

Early childhood caries (ECC) is one of the most common diseases in children under the age of six. Despite efforts toward the prevention of ECC, it affects about 621 million children worldwide [1]. The prevalence of ECC varies between different countries and regions: the lowest prevalence of 7% was found in Nigeria and the highest in Indonesia with 90% [2]. There are several risk factors for ECC, such as late commencement of tooth brushing, lack of supervision of tooth brushing by parents and consumption of sugar-containing beverages at night-time [3]. A questionnaire study conducted in Qatar revealed that the knowledge, attitudes and practices of parents regarding ECC were relatively fair [4]. Mothers demonstrated greater knowledge than fathers concerning the oral health issues of their children, and highly educated parents had better attitudes than less educated parents.

ECC is regarded as a highly prevalent but often neglected global burden [5]. Dental treatment for ECC is in many cases only possible under general anaesthesia due to the complex oral rehabilitation required and the child’s stage of cognitive development at that very young age; however, any use of general anaesthesia can bear risks for the child [6]. Among the postoperative complications following general anaesthesia dentistry, postoperative pain is the most prevalent complication [7]. Moreover, general anaesthesia also has an economic impact, leading to higher costs for the public health system. Children who require dental treatment for ECC under general anaesthesia carry a high burden of disease and they are often among those who have no access to early preventive visits [8]. Early dental visits for children are considered helpful to prevent the development of ECC [9].

The experience of early pain as a consequence of ECC affects both the immediate and long-term quality of life of children [3]. A study showed that ECC had an impact on oral health-related quality of life (OHRQoL) among Austrian preschool children and that dental treatment under general anaesthesia improved OHRQoL. After dental rehabilitation, children were able to eat, drink and sleep without experiencing toothache [10]. Both the preschool children’s OHRQoL and that of their families was improved after restorative treatment under general anaesthesia [11]. Another study showed that caries had a negative influence on OHRQoL in children aged 7 to 10 years and could lead to poor oral health when dietary and oral hygiene habits were not altered [12].

After dental rehabilitation under general anaesthesia, recall visits are necessary for children with high caries risk. Concerning the recall periodicity for children, it has been found that children who attended recalls at both three- and six-month intervals showed a greater decrease in their caries risk level compared to children who returned only at six-month intervals [13]. Two kinds of oral-health-promotion interventions were used to evaluate ECC prevention in children aged 2–5 years after dental treatment under general anaesthesia [14]. One group received fluoride varnish four times a year and an educational pamphlet containing instructions for proper oral and dietary hygiene. The other group received the same and, additionally, six reminder phone calls, scheduled for once a month during the first six months after dental treatment under general anaesthesia. Improvement in the self-reported performance of mothers was seen in both groups.

In Switzerland, a prevention program with the aim of reducing ECC was evaluated to find out if the 2-year-old children who were most in need for it had actually been reached [15]. The target group, children with high caries risk, were less likely to attend the toddler check-up. Unfortunately, parents and their children often do not attend control visits after dental treatment under general anaesthesia. These children are difficult to reach and therefore often not included in dental control visits aiming to prevent further dental caries after general anaesthesia. Thus, the aim of this study was to assess oral health in children following dental treatment under general anaesthesia and to obtain information about oral health measures in both the children and their parents.

## 2. Materials and Methods

### 2.1. Study Population

**Figure 1:**
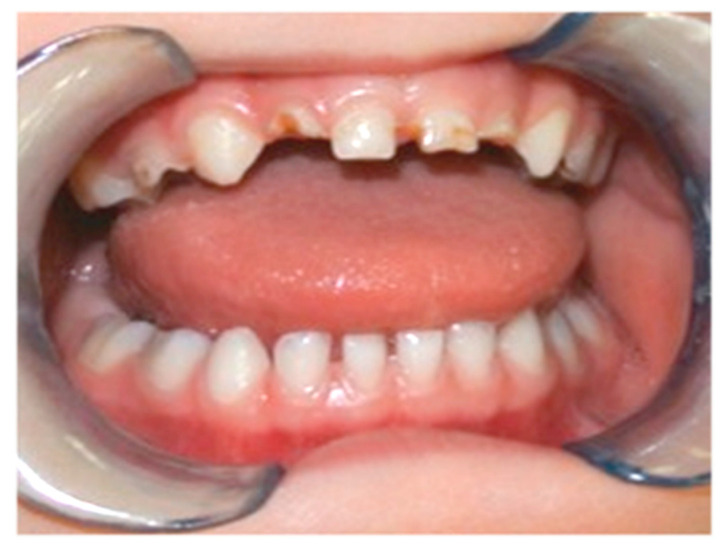
Early childhood caries affecting the primary maxillary anterior teeth of a small child.

A total of 134 children (age: 2–17 years, mean ± SD: 5.5 ± 3.3 years, median: 4 years) who had previously exhibited ECC (Figure 1) and received dental treatment under general anaesthesia at the Department of Periodontology and Operative Dentistry of the Medical Center of the Johannes Gutenberg University Mainz, Germany, were initially invited by telephone call to return for regular dental visits.

The majority (73; 54.5%) showed no interest, 15 (11.2%) could not be reached due to invalid phone numbers and 5 (3.7%) found it too far to travel from their home. Of the 41 parents/children, who agreed to a regular control visit, 6 (4.6%) did not come to the appointment. Thus, finally, only 35 of 134 children (26.1%) came for the dental re-examination. The parents accompanying the children were informed in writing about the aim of the study and signed a written consent form allowing their children to participate. All data were anonymised before analysis and can thus no longer be traced back to particular individuals. At the time of re-examination, the children’s dental treatment under general anaesthesia had occurred at least one year ago or at most six years ago. Healthy children as well as children with pre-existing conditions were included. The previous treatments under general anaesthesia had been performed only when extensive oral rehabilitation was needed and the child was uncooperative due to their cognitive development, or when several dental treatment attempts had failed. For assessment of caries, mixed dmft/DMFT index scores were determined for cavitated lesions in the primary (decayed, missing, filled teeth (dmft)) and permanent dentition (DMFT).

The dental re-examination was carried out using a dental mirror and probe. Assessment of oral hygiene was performed using the plaque control record (PCR) [16] and the gingival bleeding index (GBI) [17]. For plaque control, a child-friendly tooth rinsing solution was chosen (Plaque Agent^®^, Hager & Werken, Duisburg, Germany). For the determination of GBI, a periodontal probe was used (PCP11, Hu-Friedy, Chicago, IL, USA).

### 2.2. Questionnaire

In addition to the clinical re-examination, the parents of the children were asked to fill in a questionnaire, which contained questions about children’s/parents’ oral hygiene habits and frequencies of dental visits. The questionnaire was voluntary.

### 2.3. Statistical Analysis

The data were analysed using the statistical program SPSS version 22 (SPSS, Chicago, IL, USA) with the assistance of the Institute of Medical Biostatistics, Epidemiology and Informatics (IMBEI), University Medical Center of the Johannes Gutenberg University Mainz, Germany. For categorical variables (gender, answers to questions from the questionnaire), only absolute and relative frequencies are given here as descriptive information. For numerical continuous and discrete variables (age, PCR, GBI, mixed dmft/DMFT), the mean, standard deviation (SD), median, minimum (min), maximum (max) and frequencies were calculated. The mixed dmft/DMFT index scores as assessed before dental treatment under general anaesthesia and at re-examination were compared using the Wilcoxon test for related samples. The significance level was set at *p* < 0.5.

## 3. Results

### 3.1. Oral Health of the Children

Demographic data and oral health parameters for the participants are presented in Table 1 and Table 2.

A total of 35 children (n = 20 (57.1%) girls/n = 15 (42.9%) boys) were re-examined after dental treatment under general anaesthesia. The median age of the participants at the recall visit was 6 years (range: 4–22 years, mean ± SD: 7.8 ± 4.4 years). Of the 35 children, 18 (51.4%) were healthy, whereas 17 (48.6%) had a pre-existing condition. The majority of the latter either had some kind of cardiac disease (7 out of 35, 20%) or a developmental disorder (6 out of 35, 17.1%). The period between the first visit and the recall visit was at least 1 and no more than 6 years. For the majority of the children (24, 68.6%), this period was one (15, 42.9%) or two years (9, 25.7%).

**Figure 2:**
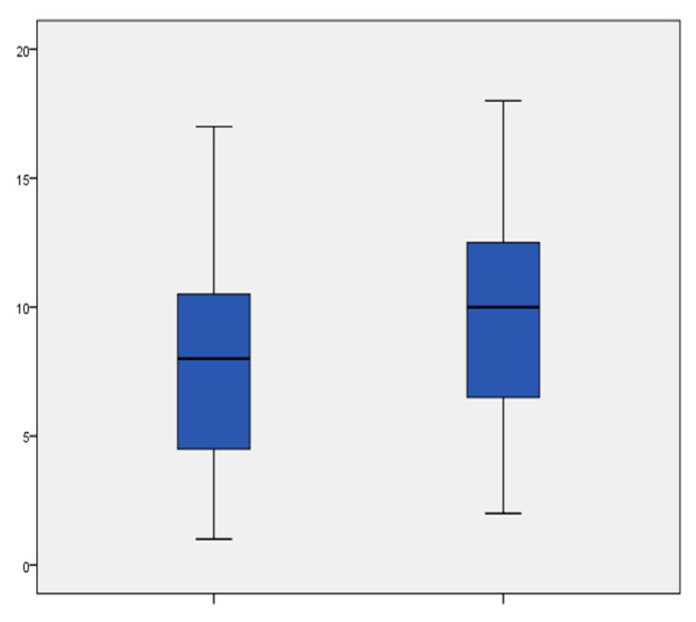
Mixed dmft/DMFT scores at the first (left box) and the recall (right box) visits. The difference was statistically significant (*p* = 0.006). Medians are shown as lines inside the boxes, 25th and 75th percentiles as boxes, maximum and minimum values as whiskers; n = 35.

Mixed dmft/DMFT scores (Figure 2) determined at the recall visit differed statistically significantly from those at the first visit (*p* = 0.006).

Concerning caries prevalence, the children exhibited 1.74 ± 3.64 teeth newly affected by caries. Thus, four children (11.4%) needed dental treatment under general anaesthesia again.

With regard to the oral hygiene assessment after dental treatment under general anaesthesia, oral hygiene was mediocre, with a median PCR of 32%, and GBI was high, with a median of 14%. Children with a high PCR also showed a high GBI.

The dental procedures performed under general anaesthesia are presented in Table 3 and included fillings, tooth extractions, fissure sealants and stainless steel crowns (SSC).

**Figure 3:**
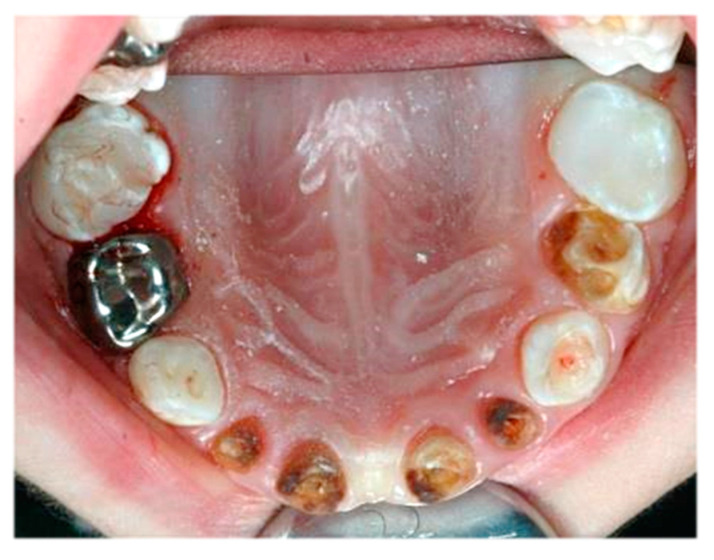
Severe early childhood caries in the primary maxillary teeth of a child requiring several types of dental treatment under general anaesthesia.

In total, 14 children (40%) only received fillings, while two or three further procedures were performed in 21 children (60%). An example of the dentition of a child requiring multiple dental procedures is shown in Figure 3.

### 3.2. Questionnaire

The oral hygiene habits and dental visits of the children and the accompanying parents are presented in Table 4.

The majority of the accompanying parents (25; 71.4%) stated that they had not changed the oral hygiene habits of their children after dental treatment under general anaesthesia.

Among the accompanying parents, 24 (68.6%) answered that they brushed their teeth twice daily, whereas 5 (14.3%) brushed their teeth three times per day and 6 (17.1%) only performed tooth brushing once a day. Twelve (34.3%) of the parents reported that they never used dental floss and thirteen (37.1%) that they seldom used it, while only three (8.6%) used it frequently and seven (20%) used it always. Twelve of the parents (34.3%) said they never used mouth rinse and eleven (31.4%) stated that they seldom used it, whereas nine (25.7%) used it frequently and only three (8.6%) always used it.

The majority of the parents (25; 71.4%) stated that their children performed tooth brushing twice daily, four (11.4%) of them reported three times per day, five of them (14.3%) once a day and one parent (2.9%) answered that their child brushed less than once per day. According to the answers of the parents, the majority of the children (29; 82.9%) never used dental floss and four (11.4%) of them seldom used it, whereas only one child (2.9%) used dental floss frequently and one child (2.9%) always. It was also reported that the majority of the children (22; 62.9%), never used mouth rinse, 11 (31.4%) seldom used it and only 2 children (5.7%) used mouth rinse frequently. Concerning the question whether or not the children performed tooth brushing on their own, it was stated that 14 (40%) of the children always brushed their teeth on their own and 10 (28.6%) did so frequently, whereas 7 (20%) seldom brushed their teeth on their own and 4 (11.4%) never did so.

The majority of the accompanying parents (27; 77.1%) stated that they brushed their children’s teeth again afterwards.

Concerning the frequency of their own dental visits, a slim majority of the parents (18; 51.4%) answered that they visited a dentist twice a year and about a quarter of them (9; 25.7%) once a year. Only five (14.3%) of the parents reported visiting the dentist less than once per year, whereas three (8.6%) of them reported quarterly dental visits. With regard to the frequency of their children’s dental visits, slightly more than half of the parents (19; 54.3%) reported quarterly dental visits, whereas 15 (42.9%) of them answered that their children saw the dentist twice a year and one (2.9%) of the parents reported only one dental visit per year. The reason for the last dental visit was a control visit for 28 children (80%), while only 2 children (5.7%) went to a dentist due to pain and 5 children (14.3%) had other reasons for their dental appointments.

## 4. Discussion

The results of the present study were based on a specific group of children, all of whom had previously been treated for ECC and high treatment needs that had to be attended to under general anaesthesia. These children were re-examined one to six years after dental treatment was performed under general anaesthesia. Children who have previously received dental treatment under general anaesthesia remain at high risk of developing new carious lesions and must return for routine dental care. However, due to uncooperative behaviour and remaining dental fear, in some cases normal dental treatment may not be possible and, therefore, further dental treatment under general anaesthesia is required.

The low response rate of 26.1% in this study can be explained by the characteristics of the specific group of children and especially their parents; the latter may be difficult to reach to invite for recall visits because of their own attitudes towards dental control visits and oral health care measures in general. Mallineni and Yiu [18] concluded in their retrospective review of outcomes of dental treatment performed for special-needs patients under general anaesthesia that the attendance at recall visits decreased from 96% to 36% within two years. The majority of the children (68.6%) in the present study were re-examined one or two years after dental treatment under general anaesthesia and the others over two years up to six years ago. It has been reported that often, after dental treatment under general anaesthesia, only patients who have pain or notice a restoration failure are willing to visit a dentist [19].

With 20 girls (57.1%) and 15 boys (42.9%), patient genders were approximately equally distributed. The median age at the time of the dental treatment under general anaesthesia was 4 years and at the recall visit it was 6 years. The age range can be explained by the fact that some of the older children had a development disorder (17.1%), which made them comparable to younger children with regard to their ability for tooth brushing and employment of other oral hygiene measures.

The mixed dmft/DMFT score of 9.6 ± 4.3 is almost identical to the mean dmft score (9.49 ± 3.51) reported by Kraljevic et al. [3] and comparable to those reported by Alkilzy et al. (8.6) [20] and Razeghi et al. (11.0 ± 4.0) [14]. In the present study, the children demonstrated a mean number of 1.74 ± 3.64 teeth newly affected by carious lesions at the recall visit, which is lower than the number reported by Almeida et al. [21], where children with ECC showed 3.2 ± 3.3 teeth newly affected by caries.

Following dental treatment under general anaesthesia, children may be at risk for repeated oral rehabilitation under general anaesthesia. In the present study, four children (11.4%) needed dental treatment under general anaesthesia again. This is in accordance with a five-year follow-up study in which 11% of the children received repeat dental treatment under general anaesthesia [22]. In the study by Almeida et al. [21], the number of children who needed further dental treatment under general anaesthesia was higher, with 17% requiring it within two years after oral rehabilitation under general anaesthesia.

Concerning dental procedures performed under general anaesthesia, 40% of the children only received fillings and 60% received fillings plus one or two other treatment procedures. Thus, the majority of treatment procedures were restorative, which is in accordance with the results of a study by Savanheimo and Vehkalahti [22] in which almost all of the children (95%) received fillings under general anaesthesia, and comparable to a study by Mallineni and Yiu [18] in which 50% of the dental treatments were restorative procedures. Fissure sealants were applied in 11.4% of the children in the present study, which is comparable to the 17% rate for fissure sealants reported as preventive procedures by Mallineni and Yiu [18] and the 21% rate for fissure sealants in the study by Savanheimo and Vehkalahti [22]. In the present study, the number of extractions was high at 81 teeth (60%), which is in accordance with 71% rate for extractions reported by Savanheimo and Vehkalahti [22]. In contrast, in the study by Mallineni and Yiu [18], a 25% rate for extractions was reported; however, in the latter study, the patients were older at the time they received dental treatment under general anaesthesia, with a mean age of 12.3 ± 10.5 years and a greater range of 1.8–50 years, and more permanent teeth were involved. In the present study, 8.6% of the children received stainless steel crowns, which is similar to the rates of 6.1% reported by Weninger et al. [8] and of 13.2% reported by Bekes et al. [23], having been placed under general anaesthesia between 2002–2011. Azadani et al. [24], in their survival analysis of primary second molars in children treated under general anaesthesia, concluded that SSCs had the highest success for cases with severe ECC. The placement of SSCs under general anaesthesia can reduce the need for further treatment because primary second molars treated, for example, with fillings are more likely to develop new caries. Jiang et al. [25] evaluated the success rates of dental procedures in children undergoing treatment for ECC under general anaesthesia and found overall high success rates for different dental treatment procedures. After six months, the success rate for SSCs was 96.3% and for resin composite restorations it was 89.6%; however, after one year, the success rate remained at 95.1% for SCCs but decreased to 78.8% for resin composite restorations.

It has been reported that children may show high plaque scores and gingival bleeding before dental treatment under general anaesthesia [20]. In the present study, children after dental treatment under general anaesthesia still exhibited plaque accumulation and gingival bleeding at the recall visit. Additional preventive appointments for children after general anaesthesia can improve their oral hygiene parameters, such as by lowering plaque accumulation and reducing gingival bleeding [20]. At the recall visits, overall oral hygiene was found to be less than optimal among the children, who exhibited only mediocre PCRs and high GBI indices. However, the majority of the accompanying parents answered that their children performed tooth brushing twice daily and that they brushed their children’s teeth again afterwards. Such statements have to be interpreted with caution, as some parents might have a tendency to give answers in a more positive way. In accordance with the present study, Kraljevic et al. [3] reported that the majority of the children they studied (71 out of 82 children) received supervised tooth brushing, which was mainly performed by their mothers. In the present study, among a total of 35 children, 14 always brushed their teeth on their own, whereas in the study by Kraljevic et al. [3], 11 out of 82 children cleaned their teeth by themselves. The majority of the children in the present study performed tooth brushing at least twice daily, which is in accordance with the study by Kraljevic et al. [3], where most children also brushed their teeth at least twice per day.

One limitation of the present study was its small sample size. The limited number of participants was the result of the voluntary participation and due to the parents’ limited willingness to attend dental control visits with their children. Another limitation of this study was the lack of a control group. Almeida et al. [21] compared 42 children with ECC with 31 control children who initially showed no carious lesions. Furthermore, there may be a potential risk of recall bias for questionnaires in general.

## 5. Conclusions

Children with ECC who had previously received various types of dental treatment under general anaesthesia were invited to recall visits taking place one to six years after the original treatment. Their oral health was assessed, and an increase in the mixed dmft/DMFT index scores was found and their oral hygiene judged to be less than optimal. These children still had a high caries risk. Therefore, better suited prophylaxis programs are necessary to prevent caries and further dental treatment under general anaesthesia.

## Figures and Tables

**Table 1:** Demographic data for the participants.

Demographic Data	Result		
Number of participants	35		
Female/male (%)	20/15 (57.1%/42.9%)		
	Mean ± SD	Range	Median
Age at first visit (years)	5.5 ± 3.8	2–17	4
Age at recall visit (years)	7.8 ± 4.4	4–22	6
Healthy	18 (51.4%), 13 female, 5 male		
With pre-existing condition	17 (48.6%), 7 female, 10 male		

**Table 2:** Oral health parameters for the participants.

Oral Health Parameters	Result		
	Mean ± SD	Range	Median
dmft/DMFT at first visit	7.9 ± 4.1	1–17	8
dmft/DMFT at recall visit	9.6 ± 4.3	2–18	10
Difference in dmft/DMFT between visits	1.74 ± 3.54	0–15	0
Plaque control record (PCR)	37 ± 22%	10–100%	32%
Gingival bleeding index (GBI)	20 ± 18%	0–79%	14%

**Table 3:** Dental procedures performed under general anaesthesia.

Dental Treatment Procedures	Fillings	Tooth Extractions	Fissure Sealants	Stainless Steel Crowns
Number of treatments	177	81	11	8
Number of children	35 (100%)	21 (60%)	4 (11.4%)	3 (8.6%)
Range	1–13	0–11	0–4	0–4
Mean ± SD	5.1 ± 3.2	2.3 ± 2.9	0.3 ± 1.0	0.2 ± 1.5
Median	4	1	0	0

**Table 4:** Oral hygiene habits and dental visits of the children and the accompanying parents.

Oral Hygiene Habits	Parents (n = 35)	Children (n = 35)
Tooth brushing		
Less than 1 × per day	0 (0%)	1 (2.9%)
1 × per day	6 (17.1%)	5 (14.3%)
2 × per day	24 (68.6%)	25 (71.4%)
3 × per day	5 (14.3%)	4 (11.4%)
More frequently	0 (0%)	0 (0%)
Usage of dental floss		
Never	12 (34.3%)	29 (82.9%)
Seldom	13 (37.1%)	4 (11.4%)
Frequently	3 (8.6%)	1 (2.9%)
Always	7 (20%)	1 (2.9%)
Usage of mouth rinse		
Never	12 (34.3%)	22 (62.9%)
Seldom	11 (31.4%)	11 (31.4%)
Frequently	9 (25.7%)	2 (5.7%)
Always	3 (8.6%)	0 (0%)
Children brush their teeth on their own	n.a.	
Never		4 (11.4%)
Seldom		7 (20%)
Frequently		10 (28.6%)
Always		14 (40%)
Parents brush the children’s teeth again afterwards		
Yes/no	n.a	27 (77.1%)/8 (22.9%)
**Dental visits**	**Parents**	**Children**
Frequency of dental visits		
4 × per year	3 (8.6%)	19 (54.3%)
2 × per year	18 (51.4%)	15 (42.9%)
1 × per year	9 (25.7%)	1 (2.9%)
Less than 1 × per year	5 (14.3%)	0 (0%)
Reason for the last dental visit	n.a.	
Control visit		28 (80%)
Pain		2 (5.7%)
Other		5 (14.3%)

## Data Availability

Not applicable.

## References

[1] Kassebaum N.J., Bernabé E., Dahiya M., Bhandari B., Murray C.J., Marcenes W. (2015). Global burden of untreated caries: A systematic review and metaregression. J. Dent. Res..

[2] Chen J., Duangthip D., Gao S.S., Huang F., Anthonappa R., Oliveira B.H., Turton B., Durward C., El Tantawi M., Attia D. (2021). Oral health policies to tackle the burden of early childhood caries: A review of 14 countries/regions. Front. Oral Health.

[3] Kraljevic I., Filippi C., Filippi A. (2017). Risk indicators of early childhood caries (ECC) in children with high treatment needs. Swiss Dent. J..

[4] Al-Jaber A.S., Al-Qatami H.M., Abed Al Jawad F.H. (2021). Knowledge, attitudes, and practices of parents on early ehildhood caries in Qatar-a questionnaire study. Eur. J. Dent..

[5] Splieth C.H., Banerjee A., Bottenberg P., Breschi L., Campus G., Ekstrand K.R., Giacaman R.A., Haak R., Hannig M., Hickel R. (2020). How to intervene in the Caries Process in children: A joint ORCA and EFCD expert Delphi consensus statement. Caries Res..

[6] Oubenyahya H., Bouhabba N. (2019). General anesthesia in the management of early childhood caries: An overview. J. Dent. Anesth. Pain Med..

[7] Zhang Q., Deng X., Wang Y., Huang R., Yang R., Zou J. (2020). Postoperative complications in Chinese children following dental general anesthesia: A cross-sectional study. Medicine.

[8] Weninger A., Seebach E., Broz J., Nagle C., Lieffers J., Papagerakis P., Da Silva K. (2022). Risk indicators and treatment needs of children 2–5 years of age receiving dental treatment under general anesthesia in Saskatchewan. Dent. J..

[9] Geiken A., Holtmann L., Splieth C.H., Conrad J., Doerfer C.E., Graetz C. (2022). Are the dental guidelines for early dental visits and fluoridation measures supported by pediatricians, and what are their caries prevention efforts?. J. Clin. Med..

[10] Boukhobza S., Stamm T., Glatthor J., Meißner N., Bekes K. (2021). Changes in oral health-related quality of life among Austrian preschool children following dental treatment under general anaesthesia. Clin. Oral Investig..

[11] Alantali K., Al-Halabi M., Hussein I., El-Tatari A., Hassan A., Kowash M. (2020). Changes in preschool children’s oral health-related quality of life following restorative dental general anaesthesia. Br. Dent. J..

[12] Michaelis L., Ebel M., Bekes K., Klode C., Hirsch C. (2021). Influence of caries and molar incisor hypomineralization on oral health-related quality of life in children. Clin. Oral Investig..

[13] Berry E.J., Brickhouse T.H., Kerns A.K., Nordeen K.A., Best A.M. (2017). Effectiveness of a preventive recall strategy for children after dental rehabilitation with general anesthesia. Pediatr. Dent..

[14] Razeghi S., Amiri P., Mohebbi S.Z., Kharazifard M.J. (2020). Impact of health promotion interventions on early childhood caries prevention in children aged 2–5 years receiving dental treatment under general anesthesia. Front. Public Health.

[15] Mühlemann A., von Felten S. (2021). Evaluation of a caries prevention programme for preschool children in Switzerland: Is the target group being reached?. BMC Oral Health.

[16] Ainamo J., Bay I. (1975). Problems and proposals for recording gingivitis and plaque. Int. Dent. J..

[17] O’Leary T.J., Drake R.B., Naylor J.E. (1972). The plaque control record. J. Periodontol..

[18] Mallineni S.K., Yiu C.K. (2014). A retrospective review of outcomes of dental treatment performed for special needs patients under general anaesthesia: 2-year follow-up. Sci. World J..

[19] Leagault J.V., Diner M.H., Auger R. (1972). Dental treatment of children in a general anaesthesia clinic: Review of 300 cases. J. Can. Dent. Assoc..

[20] Alkilzy M., Schmoeckel J., Schwahn C., Basner R., Al-Ani A., Takriti M., Splieth C. (2022). Multicenter RCT on intensive caries prevention for children undergoing dental general anaesthesia: Intensive caries prevention for children undergoing dental general anesthesia. J. Dent..

[21] Almeida A.G., Roseman M.M., Sheff M., Huntington N., Hughes C.V. (2000). Future caries susceptibility in children with early childhood caries following treatment under general anesthesia. Pediatr. Dent..

[22] Savanheimo N., Vehkalahti M.M. (2014). Five-year follow-up of children receiving comprehensive dental care under general anesthesia. BMC Oral Health.

[23] Bekes K., Steuber A., Challakh N., Schmidt J., Haak R., Hraský V., Ziebolz D. (2020). Associated factors to caries experience of children undergoing general anaesthesia and treatment needs characteristics over a 10 year period. BMC Oral Health.

[24] Azadani E.N., Peng J., Kumar A., Casamassimo P.S., Griffen A., Amini H., Ni A. (2020). A survival analysis of primary second molars in children treated under general anesthesia. J. Am. Dent. Assoc..

[25] Jiang H., Shen L., Qin D., He S., Wang J. (2019). Effects of dental general anaesthesia treatment on early childhood caries: A prospective cohort study in China. BMJ Open.

